# Decentralized control of human visceral leishmaniasis in endemic urban areas of Brazil: a literature review

**DOI:** 10.1186/s41182-016-0011-z

**Published:** 2016-04-21

**Authors:** Sonia S. Menon, Rodolfo Rossi, Leon Nshimyumukiza, Kate Zinszer

**Affiliations:** International Centre for Reproductive Health (ICRH), Ghent University, De Pintelaan 185 P3, 9000 Ghent, Belgium; Department of Epidemiology and Biostatistics, McGill University, Montreal, Canada; LSHTM alumni, Beirut, Lebanon; Department of Social and Preventive Medicine, Laval University, Boston, USA; Boston Children’s Hospital, Harvard Medical School, Boston, MA USA

**Keywords:** Human visceral leishmaniasis, HIV, Control and prevention, Decentralization, Human reservoir, Basic reproductive number, Visceral leishmaniasis, HIV co-infection

## Abstract

**Objectives:**

Human migration and concomitant HIV infections are likely to bring about major changes in the epidemiology of some parasitic infections in Brazil. Human visceral leishmaniasis (HVL) control is particularly fraught with intricacies. It is against a backdrop of decentralized health care that the complex HVL control initiatives are brought to bear. This comprehensive review aims to explore the obstacles facing decentralized HVL control in urban endemic areas in Brazil.

**Method:**

A literature search was carried out in December 2015 by means of three databases: MEDLINE, Google Scholar, and Web of Science.

**Results:**

Although there have been many strides that have been made in elucidating the eco-epidemiology of *Leishmania infantum*, which forms the underpinnings of the national control program, transmission risk factors for HVL are still insufficiently elucidated in urban settings. Decentralized HVL epidemiological surveillance and control for animal reservoirs and vectors may compromise sustainability. In addition, it may hamper timely human HVL case management. With the burgeoning of the HIV-HVL co-infection, the potential human transmission may be underestimated.

**Conclusion:**

HVL is a disease with focal transmission at a critical juncture, which warrants that the bottlenecks facing the control program within contexts of decentralized healthcare systems be taken into account. In addition, HIV-driven HVL epidemics may substantially increase the transmission potential of the human reservoir. Calculating the basic reproductive number to fine-tune interventions will have to take into consideration the specific socio-economic development context.

## Background

Human visceral leishmaniasis (HVL) is endemic in 70 countries [1]. *Leishmania infantum chagasi* is the main species causing HVL in Brazil. It is transmitted by sandflies, the species *Lutzomyia longipalpis* being the most important HVL vector in the New World. The parasite *L. infantum* multiplies within the sandfly for a period between 8 and 20 days and, in humans, it has an incubation period of between 2 and 6 months [2]. In Brazil, canines are the main reservoirs [3], with humans not considered necessary for maintaining transmission within the community. A recent mathematical model demonstrated that the insecticide-impregnated dog collars and vector control [4] were the most effective interventions in reducing the prevalence of HVL in humans.

The HVL disease burden in Latin America is unknown as most countries lack effective surveillance systems, resulting in substantial underreporting [5–7], Brazil, which harbors 90 % of the VL cases documented on the American continent, [8] registered 70 thousand cases of HVL between 1980 and 2008 resulted in the deaths of more than 3800 people [9, 10].

The typical manifestations of HVL include fever, weight loss, hepatosplenomegaly, and pancytopenia resulting from replication of *Leishmania* amastigotes in macrophages mainly in the liver, spleen, and bone marrow, causing severe and ultimately lethal lesions [11]. Typical features such as splenomegaly may be absent in VL-HIV-co-infected patients [12], whereas atypical organ involvement, such as of the lungs or gastrointestinal system and renal failure has been associated with chronic VL in HIV patients [13–15]. Conversely, as HIV viral load increases in patients with HIV-*Leishmania* co-infection [16], it promotes the clinical progression of HIV and the development of AIDS-defining conditions [17].

HVL was formerly restricted to rural areas in Brazil, but since the 1980s, it has been spreading to urban centers. This spread has also lead to increasing numbers of HIV co-infected cases [18], as HIV is also endemic in urban centers in Brazil [19]. In 1988, Brazil adopted a new federal constitution that called for a nationally unified health system and facilitated the process of municipalization. In this process, municipal governments, the smallest autonomous political and geographic unit within the federal system, took on increasing levels of resources and responsibility for a range of health and other services, based in part on evaluations of their managerial capacities [20].

From the 1950s to the end of the 1990s, the Brazilian VL program was the responsibility of the federal government. As result of the decentralization of the control programs for epidemics, the control of leishmaniasis has become the responsibility of the municipality [21, 22]. The decentralization of the program to the states and municipalities has been implemented amidst difficulties at these levels of governments, which have insufficient accumulated experience in control [23]. Control programs over the past 20 years in Brazil have been accompanied by a substantial decentralization process [24], which has been attributed to the successful control of cholera, Chagas, and vaccine-preventable diseases [10] but unsuccessful for certain vector-borne diseases including HVL and dengue fever, both vector-borne diseases with changing epidemiological profiles [10].

The objectives of this review were to highlight the potential shortcomings of a HVL control program in a decentralized context and to identify the clinical and epidemiological research gaps for HVL control and prevention.

## Methods

A literature search was carried out in December 2015 by means of three electronic databases: MEDLINE (1948-December 2015), Google Scholar, and Web of Science (1899-December 2015). Papers in English and Portuguese were identified using medical subject headings and truncations

Control of the animal reservoir and arthropod vector: (visceral leishmaniasis OR *Leishmania chagasi* OR *L chagasi* OR Kala-azar *Leishmania infantum OR* phlebotomine sandflies) AND “Brazil” AND control.

Diagnosis of human VL: (visceral leishmaniasis OR kala-azar OR *L.infantum OR L. chagasi OR Leishmania infantum OR Leishmania chagasi*A ND (diagnostic accuracy OR sensitivity OR specificity AND DAT OR dipstick) AND “Brazil”

Treatment of human VL: For the PubMed search the following key-words were used: (visceral leishmaniasis *L. chagasi* AND treatment OR amphotericin B OR Amphotericin B deoxycholate OR pentavalent antimonials OR) AND (HIV positive OR AIDS) AND “Brazil”

Diagnosis of canine VL: (canine visceral leishmaniasis OR *L.infantum OR L.chagasi OR L.donovani OR Leishmania infantum OR Leishmania chagasi)*AND (diagnostic accuracy OR diagnostic performance OR sensitivity OR specificity OR validation) AND “Brazil”

Decentralization of health care: (decentralization AND vector-borne diseases) AND Brazil OR (decentralization of health care AND Brazil)

An additional manual search was undertaken to include the bibliographies of the retrieved references.

## Results

### Epidemiology

Human visceral leishmaniasis (HVL) is a disease of public health importance caused by protozoans belonging to the genus *Leishmania*, which is present worldwide, particularly in Brazil, Bangladesh, India, Nepal, and Sudan [25]. The humans, animal reservoir, and vector share the same ecological niche, which contributes to the persistence of the disease. There is a consensus that the control of phlebotomine sandflies, the vector, is a daunting task as the identification of their breeding sites is challenging, which limits the effectiveness of control measures focused on immature forms of these vectors [26]. In Brazil, sandflies have spread in both rural and urban area, which contrasts with most other New World eco-epidemiological situations where sandflies are associated with forested areas.

Infectious dogs are considered the main reservoir and are estimated to have a basic reproduction number (*R*_0_) of 6, meaning that each infected dog gives rise to an average of six new cases [27]. A study found that in the city of Petrolina (State of Pernambuco, northeast region, Brazil), of the 600 dogs tested, 19 % presented anti-*L. infantum* antibodies [28]. The transmission of *L. infantum*, which was restricted to rural areas and spread in the 1980s to urban areas [9], is of great public health concern and is the result of the environmental changes, human disease reservoir migration, and adaptation of the sandflies vector to the peri-domiciliary [29]. In the last 20 years, despite the known underestimation of cases, Brazil registered a marked increase in the incidence of visceral leishmaniasis [27]. Whilst in the 1980s, HVL was considered a rural disease, HVL has spread throughout municipalities with 7 % of municipalities being endemic in 1985; 18 % in 1990; 30 % in 1996 and, in 2014, endemicity was reported in 21 of the 26 Brazilian states plus the Federal District [30] and its control in urban areas constitutes a challenge [31]. In the 1980s, an average of 1500 cases was reported each year in Brazil, and between 2000 and 2009, the average increased to 3480 cases annually, an increase of 132 % [32]. During the period of 2002–2009, case fatality rate varied between 8.5 % in 2003 and 5.6 % in 2008, with an average of 7.0 %, compared to 3.2 % in 1994 [33]. However, case fatality rates derived from the national Database on Reportable diseases (SINAN) are subject to an underreporting of 45.5 % [34] depending on the municipality, due to deficiencies in access to health services and quality of care along with atypical clinical manifestations [35].

Of the 44,289 cases reported in Brazil by SINAN during 1980 to 2000, 89.9 % of these came from the northeast. Among all the Federal states in Brazil, Maranhão, a state in northeastern Brazil has recorded the highest number of cases of HVL. Between 2000 and 2009, 5389 cases of HLV were registered, with the highest incidence in the Regional health unit of Caxias, which reported 36.1 cases per 100,000 inhabitants [36].

In Northeastern Brazil, HIV has also become increasingly prevalent [37]; however, information on co-infection by VL and HIV in the northern region of Brazil is still scarce [38]. HIV-HVL co-infection has become an emerging public health issue [39]. The urbanization of HVL has resulted in a geographical overlap between HVL and AIDS in Brazilian inner cities [40].

### Diagnostics

According to the Brazilian Ministry of Health, a diagnosis of HVL requires the identification of the parasite in a smear or culture and/or positive serological testing in patients presenting with fever and spleen enlargement [41]. The once widely used serological test for HVL diagnosis in Brazil immunofluorescence (IFI) [42], which was typically performed in referral laboratories around the country and required a delay of a few days to obtain the results [43], has been replaced by the use of rapid diagnostic tests (RDTs). This has been introduced to circumvent mortality by reducing diagnostic delay, as RDTs have been shown to improve the early detection of HVL, but their real-world performance in large urban settings requires additional study [34]. In Brazil, estimates of the sensitivity and specificity of RDTs vary between 85.7 and 100 % and 82.0 and 100 %, respectively [44–47], although only a few studies have been conducted outside of controlled settings [48]. Additionally, RDTs have a low sensitivity for HVL among HIV-infected patients, estimated at 60 % (95 % CI 40.7–76.6 %). This low sensitivity results in many undetected cases and therefore requires invasive parasite detection to diagnose atypically localized manifestations of VL [49]. The invasive nature of the tests, including the parasitological demonstration of the parasite in tissue smears requires considerable expertise [50] and carries the risk of potentially fatal bleeding. Due to high treatment failure and the relapsing nature of the disease, HIV-HVL patients are repeatedly exposed to these tests.

### Treatment

The first drug for the treatment of leishmaniasis is pentavalent antimonial, which presents high toxicity and exhibits a recurrence rate of 20 to 45 % [51–54]. The immune deficiency caused by HIV facilitates the multiplication of the *Leishmania* parasite and further reduces cure rates through conventional treatments [55–58]. In addition, HIV-HVL have higher rates of drug toxicity, higher mortality rates, resistance to pentavalent antimonial compounds, [59] and more relapses, especially if CD4+ counts are <200 cells/μl, when compared with HIV-negative VL patients [60]. The unfavorable outcomes of co-infected patients prompted the Brazilian Health Ministry to revise therapeutic guidelines in 2013 and recommended HIV co-infection as an indication for therapy with liposomal amphotericin, which is more expensive and has a reduced toxicity as well as a high level of efficacy with a 90 % cure rate [61]. Overlapping HIV and HVL prevalence increases the risk of a HVL outbreak and possibly a drug-resistant one [62].

Concomitant HIV infection increases the risk of developing active HVL by between 100 and 230 times [63], and AIDS also disproportionately affects the Brazilian urban poor [64]. Coupled with the change in transmission HVL pattern from the countryside to cities, this has led to a surge of epidemic of *L. infantum*-HIV co-infections in urban areas [65]. A recent ecological study identified high-risk areas of human HVL cases, with most clinical cases being among children and as opportunistic infections in HIV-infected patients [66] in the northern part of Belo Horizonte, the capital of the state Minas Gerais. This high-risk area also had a higher prevalence of poverty and a higher number of infected dogs per inhabitants than other parts of the city [67]. Moreover, as parasitemia is frequent in HIV-HVL [68], co-infected patients may be highly infectious to phlebotomine sandflies [69], leading to increased HVL within the community.

### Surveillance and control strategies

The Brazilian National System for Surveillance and Control of Diseases is a decentralized, hierarchical, and integrated network that uses a horizontal approach, which attempts to reduce other vector-borne diseases along with a broad-based participatory approach, as part of the Unified Health System [70]. The backbone of the national HVL Control and Surveillance Program is to reduce the morbidity and case fatality rates through early diagnosis and treatment of human cases. Moreover, it sets to curb the transmission risk by means of controlling the population of both domestic reservoirs and the vector [71]. The program coordinates (i) vector population control by means of residual insecticide spraying and environmental management and (ii) culling of seropositive dogs in areas with moderate to high levels of transmission [72]. This approach lies upon the assumption that the incidence of *L. infantum* infection in humans is directly related to the number of infectious dogs and the vector capacity of the sandfly population to transmit infection from dogs to humans [73]. Epidemiological surveillance of HVL, insecticide spraying, and dog culling control activities have been decentralized to municipalities, which since 1994, have been given responsibility to plan and provide these services [74].

### Program limitations

The decentralization program was initially successful in putting in place a large-scale deployment of insecticide spraying and dog culling [75]. However, the spread of HVL to urban settings after more than 40 years of large-scale deployment of insecticide spraying and dog culling prompts an urgent revision of the Brazilian HVL control program [76], since both strategies have obtained limited results in interrupting transmission [61, 76, 77]. Since the mid-eighties, studies have reported HVL in large urban centers, including the southeastern metropolitan areas of São Paulo [78], Rio de Janeiro [79], and Belo Horizonte [80, 81] as well as in the northeastern capitals of Teresina [74], São Luis [82, 83], and Fortaleza [84]. A systematic review of studies conducted in Latin America underscores the lack of scientific evidence to support the effectiveness of such interventions [85], and also highlights the need to address the lack of political commitment, gaps in scientific knowledge, and poor case management and surveillance systems [77].

The decentralization of epidemiological surveillance and HVL control activities to the municipalities has highlighted deficiencies in infrastructure at the local level for addressing the complexity of HVL control [86]. A recent non-significant effect of insecticide spraying on the incidence of human infection was determined for a community randomized controlled trial in Teresina, the capital of the Brazilian state of Piauí, in northeast Brazil. It illustrated how the shortage of available equipment and trained personnel for large-scale spraying interventions hampers the sustainability of control actions [69, 84, 87–90].

Although dog culling appears to have been effective in reducing infection among humans in China [91], in Brazil, where infectious dogs have been estimated to have an *R*_0_ of approximately 6 [92], HVL has surged in the past two decades despite the spraying of 200,000 houses and killing of 20,000 seropositive dogs per year [93]. A mathematical model estimated that killing two thirds of the infected dog population would result in less than 20 % reduction in the incidence of human disease [94]. Furthermore, immunofluorescence (IFI) antibody tests commonly used for mass screening of dogs [95] has low sensitivity and specificity [96], with estimates for sensitivity ranging from 72 to 100 % and for specificity from 52 to 100 % [84]. However, newer sensitive molecular diagnostic methods for canine visceral leishmaniasis, like the conjunctival swab (CS) real-time PCR reported that among the 1350 dogs screened, 369 (27.3 %) were positive by CS real-time PCR and 126 (9.3 %) tested positive by serological assays, which demonstrate its potential as a mass screening tools in endemic settings to contribute to disease control [97].

A recent simulation study in Brazil indicated that very low transmission settings (3 % prevalence), culling of only symptomatic dogs, is sufficient to maintain prevalence under 1 %. In higher endemicity settings (*R*_0_ = 1.29, prevalence = 15 %) [98], removing clinically diagnosed dogs, would be insufficient as a control strategy given that the asymptomatic population of dogs would be significant enough to maintain transmission [75, 87, 99–101].

The failure of dog culling to reduce human cases may also suggest the possibility of other reservoirs [102]. A recent serological survey performed in Petrolina showed that cats had a prevalence of 3.9 % of HVL [103].

Studies have shown that humans, crab-eating foxes, opossums, domestic cats, and black rats can also transmit *L. infantum* to sandflies; nevertheless, their importance is deemed to be minimal [77]. However, in certain scenarios these secondary reservoirs would be capable of sustaining transmission, which would warrant further studies on their transmission potential [104].

### HVL clinical management within a decentralized context

The decentralization of health care to the municipality level also has had an impact on the early detection and effective clinical management of HVL. A study in northwestern Paraná State revealed that both early treatment initiation and clinical evaluation were more complete with a centralized healthcare system than with a decentralized one [105]. After the decentralization, 32 % of patients were treated with the first line drug, pentavalent antimonial, and were treated without compliance to the recommended dosage of 30 days at 20 mg/kg/day [106]. Furthermore, 73 % of patients in the study were noted to having received inadequate treatment, and 84 % of patients failed to receive proper clinical follow-up [106]. The lack of rigorous clinical management ensuing decentralization may have an even more profound effect for clinical management of co-infection HIV-HVL, in whom HVL appears in atypical forms and is more difficult to diagnose [107].

## Knowledge gaps

### Active surveillance

The cost effectiveness of implementing an active surveillance system to detect HVL cases in endemic urban areas needs to be evaluated. Active surveillance in urban areas would entail involving community health workers in active case finding in the community in order to detect those patients who may not seek treatment in health facilities so that a more prompt treatment is initiated; this could be done through the use of RDT. Active case finding would enable more accurate estimates of HVL in urban areas to allow a better understanding of the actual burden. Additionally, active case finding would provide the data needed to the reliable estimates of HIV-HVL co-infection, which is currently lacking in northeast Brazil. In 2012, HIV-HVL co-infection prevalence was 8.5 % of all HVL cases [108]; however, there are likely a significant number of asymptomatic co-infected individuals which are undetected [109, 110]. The use of different diagnostic tests for active surveillance should also be evaluated, to understand the health and cost implications of differing technologies.

### Identification of other potential reservoirs and transmission risk factors

In zoonotic disease control, early diagnosis and treatment is essential for the patient, but is believed to have limited impact on transmission if the main animal reservoir or insect vectors are not targeted [111]. In Brazil, transmission risk factors for HVL are still insufficiently elucidated, notably in urban and densely populated areas [112]. The transmission potential by asymptomatic canine as well as the identification of other potential reservoirs needs to be determined. In the Indian continent, mathematical modeling has suggested that *Leishmania donovani* in asymptomatic individuals may also be an instrumental in maintaining transmission in endemic communities [113]. Mathematical modeling is also needed to determine whether *L. infantum* in asymptomatic individuals are also instrumental in maintaining transmission in endemic communities.

### Effectiveness of interventions

A systematic review of studies conducted in Latin America underscores the lack of scientific evidence to support the effectiveness of such interventions [84], and also highlights the need to address the lack of political commitment, gaps in scientific knowledge, and poor case management and surveillance systems [72]. Regular monitoring of human, vector, and reservoir VL at municipality level is vital to guide and tailor the HVL control strategy in urban centers.

### Impact of the decentralization on HVL surveillance and control interventions

There is limited research on the impact of decentralization on HVL surveillance, sandfly control, dog culling, culling of seropositive dogs, and case management. There should be a comprehensive review, which will identify the deficiencies in the system, such as bottlenecks in service provision or resources to appropriate implement a program.

### HIV-related gaps

#### Clinical management of HIV-HVL co-infection in Brazil

The recommendation to treat HIV-infected patients with amphotericin B was based on the opinion of experts and from clinical trials mostly conducted in India [114]. In East Africa, cure rates have had limited results, with 56 % of VL relapse cases demonstrating parasitological failure in Northern Ethiopia [115], and in Mediterranean countries [116]. Its efficacy in Brazil has yet to be established, and this recommendation should be further examined in the Brazilian context.

### Impact of HAART on HVL clinical management

HVL relapses have been shown to occur in patients on highly active antiretroviral therapy (HAART), despite increasing CD4+ counts and undetectable HIV loads [117, 118]. Moreover, HVL also seems to hamper the immunological recovery of the HIV-positive patients treated with HAART [119]. It remains to be investigated whether earlier CD4 count initiation may prevent HVL relapses.

### Impact of HAART on drug-resistant HVL

In light of potential HIV-driven drug-resistant HVL outbreaks [120], the prevalence of drug-resistant HVL in HIV-infected persons and immuno-epidemiological parameters should be determined. Moreover, the impact of earlier HAART initiation on preventing drug-resistant transmission should be HVL explored.

### Impact of HAART on the human reservoir

HAART may also lead to asymptomatic carriers [121, 122] and these may pose a risk for transmission in areas where the sandfly vector is present [123]. Research will need to be conducted to assess how changes in the HAART landscape, such as the WHO 2013 recommendation to initiate treatment at CD4 >350 count [124], may contribute to maintaining transmission in endemic communities.

## Conclusions

There is evidence that suggests the current practices of HVL control in Brazil have not been effective at controlling the spread of HVL. The lack of control effectiveness may be attributed to deficiencies in infrastructure at the local level for addressing the complexity of HVL control, in addition to the decentralization of health care to the municipality level, as well as clinical and epidemiological knowledge gaps.

Whilst decentralization of health care in Brazil has enhanced community participation [125], early diagnosis and treatment of human cases has also been impacted negatively by decentralization, with poor adherence of the standard treatment being reported in first-line treatment in resource-poor municipalities. Municipalities may lack the high level of clinical expertise to deal with the challenges that HIV-HVL co-infection management entail. These include suboptimal sensitivity of point of point-of-care diagnostics that require reliance on more invasive tests for monitoring lower cure rates, higher drug toxicity, drug interaction with HAART, relapse and mortality rates than those without HIV [126].

With cases of HIV-HVL co-infection burgeoning, the human transmission potential may be underestimated. As HIV disproportionately affects the Brazilian urban poor [127], who in turn are more at risk of living in poor municipalities and being co-infected with HVL, poor municipalities may fuel both a HIV-HVL epidemic and drug-resistant HVL transmission.

HVL should be considered as a disease with focal transmission [107], in which the conditions for transmission depend on local socio-economic factors. Despite the milestones in the HVL and in the canine HVL diagnostic landscape, which would enable rapid diagnoses at local level, a balance must be drawn between government and community involvement for control initiatives if local HVL control programs are to be sustained.

## Abbreviations

HAART: highly active antiretroviral therapy
HIV: human immunodeficiency virus
HVL: human visceral leishmaniasis
